# Antimicrobial Activity of Geranium Oil against Clinical Strains of *Staphylococcus aureus*

**DOI:** 10.3390/molecules170910276

**Published:** 2012-08-28

**Authors:** Monika Bigos, Małgorzata Wasiela, Danuta Kalemba, Monika Sienkiewicz

**Affiliations:** 1 Medical and Sanitary Microbiology Department, Medical University of Lodz, Hallera Sq. 1, Lodz 90-647, Poland; Email: monika.bigos@umed.lodz.pl (M.B.); malgorzata.wasiela@umed.lodz.pl (M.W.); 2 Institute of General Food Chemistry, Lodz University of Technology, Stefanowskiego Str., 4/10, Lodz 90-924, Poland; Email: danuta.kalemba@p.lodz.pl; Tel./Fax: +48-42-631-3428

**Keywords:** antibacterial activity, geranium oil, MIC, multidrug resistant strains, *Staphylococcus aureus*

## Abstract

The aim of this work was to investigate the antibacterial properties of geranium oil obtained from *Pelargonium graveolens* Ait. (family *Geraniaceae*), against one standard *S. aureus* strain ATCC 433000 and seventy clinical *S. aureus* strains. The agar dilution method was used for assessment of bacterial growth inhibition at various concentrations of geranium oil. Susceptibility testing of the clinical strains to antibiotics was carried out using the disk-diffusion and E-test methods. The results of our experiment showed that the oil from *P. graveolens* has strong activity against all of the clinical *S. aureus* isolates—including multidrug resistant strains, MRSA strains and MLS_B_-positive strains—exhibiting MIC values of 0.25–2.50 μL/mL.

## 1. Introduction

Bacteria that belong to the *Staphylococcus* genus constitute one of the most important epidemiological problems of contemporary invasive medicine. *S. aureus* has the strongest virulence potential among all the staphylococcal species. It may become a part of human bacterial flora (*S. aureus* nasal carriage) but increases the risk of the infection development, both nosocomial and community-acquired [1,2,3]. Since 1960 methicillin-resistant *S. aureus*, MRSA, has become one of the key pathogens responsible for health care associated infections that are usually difficult to treat [4]. There are a few reasons why *S. aureus* strains are able to exist persistently within the hospital environment. Their simultaneous resistance to several groups of antimicrobial agents including beta-lactam antibiotics, aminoglycosides, lincosamides, tetracyclines, and also quinolones and rifampin, seriously restricts the possible ways to treat staphylococcal infections [5]. Glycopeptides are then the drugs of choice, but *S. aureus* strains, especially methicillin resistant ones, may lose susceptibility to vancomycin (full or intermediate resistance to vancomycin) [6]. Patients belonging to the extreme age groups, immunocompromised and critically ill, usually need increasingly invasive methods of the diagnostics and therapy, what facilitates the infection development, mainly if they are or become colonized with *S. aureus* strains [7,8]. In consequence, the resulting increased morbidity and mortality as well as the extended hospital stays have an adverse influence on the whole hospital budget [9].

Essential oils derived from aromatic plants have many biological properties and can be used to prevent and treat human systemic diseases, including infectious diseases. They have been reported as exceptionally good therapeutic agents for chemoprevention, cancer suppression, antidiabetic activity and lowering serum cholesterol and triglycerides [10]. Many of them have the high activity against Gram-positive and Gram-negative bacteria, as well as against viruses and fungi. Due to their content of anti-infective agents essential oils may be applied in the fighting against the drug-resistant bacteria and for prevention of the resistance formation of pathogenic microbes. Essential oils, mainly from plants of the *Lamiaceae* and *Apiaceae* families, have been added to food, not only as flavouring agents but also as preservatives [11,12,13]. 

The *Pelargonium* (*Geraniaceae*) genus is represented by many essential oil producing species *inter alia*: *P. graveolens*, *P. odoranissimum*, *P. zonale* and *P. roseum*. Geranium oil is obtained from leaves, flowers and stalks by steam or hydrodistillation. The therapeutic effects of the oil find application in the treatment of dysentery, diarrhoea, biliary conditions, gastric ulcers, diabetes, cancer and skin diseases. The main constituents responsible for biological activity are citronellol, geraniol, linalool, isomenthone, nerol and citronellyl formate [14,15,16]. Due to these components the essential oil from *P. graveolens* has a strong antibacterial effect, with low Minimal Inhibitory Concentration (MIC) values against *S. aureus* (0.72 mg/mL), and also against *Bacillus cereus* (0.36 mg/mL) and *B. subtilis* (0.72 mg/mL) [17]. Interestingly, the possibility to use the combinations of different oils or combinations of oils with antibiotics can be also a supplementary therapy. It was found that geranium oil has the ability to reduce the antibiotic effective MIC with norfloxacin against standard *S. aureus* strains.

The overuse of antimicrobial chemotherapeutic agents, unfortunately typical of modern medicine, is evident and cannot be glossed over in silence. Thus the search for effective and safe medicines that could be used to treat staphylococcal infections is on. We have decided to determine if the essential oil derived from *Pelargonium graveolens* Ait. has antibacterial properties against clinical *S. aureus* isolates, what could make it an alternative or complementary to antistaphylococcal prophylactics or antibiotic therapy.

## 2. Results

### 2.1. Chemical Composition of Geranium Oil

The analysis of the tested essential oil derived from *Pelargonium graveolens* Ait. revealed that citronellol (26.7%) and geraniol (13.4%) were the main components. Among sixty seven constituents identified in the geranium oil other prevailing compounds like nerol (8.7%), citronellyl formate (7.1%), isomenthone (6.3%), linalool (5.2%), and 10-*epi*-γ-eudesmol (4.4%) were present. The whole chemical composition of the tested oil is shown in Table 1.

**Table 1:** Chemical composition of geranium oil of *Pelargonium graveolens* Ait.

Number	Compound	% (relative)	RI
1	α-Pinene	0.7	929
2	β-Pinene	tr	979
3	Myrcene	0.1	983
4	Car-2-ene	tr	986
5	α-Phellandrene	0.1	996
6	*p*-Cymene	0.1	1,012
7	β-Phellandrene	tr	1,020
8	Limonene	0.2	1,021
9	(*Z*)-β-Ocimene	tr	1,028
10	(*E*)-β-Ocimene	0.1	1,039
11	*cis*-Linlool oxide (f)	0.3	1,058
12	*trans-*Linlool oxide (f)	0.1	1,072
13	Terpinolene	tr	1,079
14	**Linalool**	**5.2**	1,086
15	*cis*-Rose oxide	1.4	1,097
16	*trans*-Rose oxide	0.6	1,113
17	α-Cyclogeraniol	tr	1,127
18	Isopulegol	0.1	1,130
19	Menthone	1.6	1,133
20	**Isomenthone**	**6.3**	1,144
21	Isomenthol	0.1	1,168
22	α-Terpineol	0.3	1,173
23	Estragole	0.1	1,177
24	**Citronellol**	**26.7**	1,217
25	**Nerol**	**8.7**	1,220
26	**Geraniol**	**13.4**	1,243
27	Geranial	1.1	1,246
28	**Citronellyl formate**	**7.1**	1,261
29	Neryl formate	0.1	1,264
30	Geranyl formate	2.5	1,283
31	Bicycloelemene Citronellyl acetate	0.4	1,334
32	α-Cubebene	0.2	1,349
33	Geranyl acetate	0.4	1,361
34	α-Copaene	0.5	1,377
35	β-Bourbonene	1.1	1,385
36	1,5-di-*epi*-Bourbonene	0.2	1,388
37	α-Gurjunene	0.1	1,411
38	β-Caryophyllene	1.5	1,419
39	Citronellyl propionate	0.3	1,425
40	β-Copaene	0.2	1,428
41	Guaia-6,9-diene	0.3	1,439
42	4aH,10aH-Guaia-1(5),6-diene	0.1	1,442
43	4bH,10aH-Guaia-1(5),6-diene	0.5	1,445
44	Geranyl propionate	1.0	1,452
45	Alloaromadendrene	0.2	1,459
46	7aH,10bH-Cadina-1(6),4-diene	0.2	1,469
47	γ-Muurolene	0.1	1,471
48	Germacrene D	1.0	1,477
49	γ-Selinene	0.1	1,479
50	β-Selinene	0.2	1,482
51	Bicyclogermacrene	0.7	1,491
52	α-Muurolene	0.2	1,496
53	Dihydroagarofuran	0.1	1,500
54	γ-Cadinene	0.6	1,509
55	*trans*-Calamenene	0.3	1,510
56	δ-Cadinene	0.9	1,515
57	Zonarene	0.2	1,518
58	Cadina-1,4-diene	0.1	1,525
59	Selina-4(15),7(11)-diene	0.2	1,530
60	Geranyl butyrate	1.4	1,537
61	Phenylethyl tiglate	0.7	1,554
62	Geranyl isovalerate	0.1	1,582
63	**10-*epi*-γ-Eudesmol**	**4.4**	1,613
64	γ-Eudesmol	0.1	1,620
65	Geranyl tiglate	1.0	1,675
66	Geranyl ester I	0.2	1,694
67	Geranyl ester II	0.1	1,730

RI—Retention Index; tr < 0.05%.

### 2.2. Susceptibility to Antibiotics among the Clinical *S. aureus* Strains

Overall, drug susceptibility of 70 *S. aureus* strains was analysed. Bacterial isolates came from various clinical materials: swabs from the nasal cavity—eight strains (Table 2), skin lesion—nine strains (Table 3), postoperative wounds—eight strains (Table 4), intubation tube—14 strains (Table 5), conjunctival sack—six strains (Table 6), throat—11 strains (Table 7), and from the stools—14 strains (Table 8). The detailed susceptibility to the tested antimicrobial agents of all the clinical strains is shown in the seven tables shown below. Resistance to methicillin was found in 31 bacterial isolates (44.3%) in comparison to 39 methicillin-susceptible strains (55.7%). These results were fully confirmed by E-tests: the minimal inhibitory concentration (MIC) value for cefoxitin ranged from 8 µg/mL to 256 µg/mL for MRSA and from 1.5 µg/mL to 3 µg/mL for MSSA isolates. Most MRSA strains were isolated from the nasal cavity (six out of eight strains), intubation tubes (10 out of 14 strains), and the throat (four out of 11 strains) (Table 2, Table 5 and Table 7). The majority of bacterial strains proved to be resistant to penicillin (57 strains, 81.4%). Nineteen of them turned out to be resistant only to this antibiotic. As expected, MRSA strains showed considerably higher resistance to drugs other than β-lactam antibiotics comparing to methicillin sensitive *S. aureus* strains (MSSA): 24 strains and 10 strains, respectively. Full resistance to β-lactam antibiotics as the only mechanism of drug resistance was confirmed in seven bacterial isolates (10.0%). Resistance to ciprofloxacin, gentamicin, tetracycline, and chloramphenicol was found in five strains (7.1%), six strains (8.6%), nine strains (12.9%), and four strains (5.7%), respectively. One *S. aureus* isolate displayed intermediate susceptibility to tetracycline. Twenty six *S. aureus* strains (37.1%) were simultaneously resistant to erythromycin and clindamycin, showing inducible or constitutive resistance to macrolide-lincosamide-streptogramin B (MLS_B_ resistance): 14 and 11 strains, respectively, while only one bacterial isolate displayed both these mechanisms. Most MLS_B_-positive strains were methicillin-resistant: inducible MLS_B_ resistance was found in nine MRSA strains, whereas constitutive MLS_B_ mechanism was present in 11 MRSA strains. The tested *S. aureus* strains were entirely susceptible to tigecycline, rifampin, trimethoprim-sulphamethoxazole, linezolid, fusidic acid, quinupristine-dalfopristine, vancomycin (MIC < 2µg/mL), and daptomycin (MIC < 1 µg/mL). Ten bacterial isolates (14.3%) proved to be susceptible to all the antistaphylococcal agents studied.

**Table 2:** Characteristics of *S. aureus* strains isolated from the nasal cavity.

**No.**	**MIC of geranium essential oil** **[µL/mL]**	**Susceptibility to antibiotics**																	**Total**		
		FOX MIC [µg/mL]		P	CIP	CN	E	DA	QD	TE	TGC	C	FD	LZD	RA	VA	DPC	SXT	R	I	S
1.	0.75	R	24	R	S	S	R	R	S	S	S	S	S	S	S	S	S	S	4	0	12
2.	1.75	R	24	R	S	S	R	R	S	S	S	S	S	S	S	S	S	S	4	0	12
3.	0.25	S	2	R	S	S	R	R	S	S	S	S	S	S	S	S	S	S	3	0	13
4.	1.00	R	16	R	S	S	R	R	S	S	S	S	S	S	S	S	S	S	4	0	12
5.	1.50	R	8	R	S	S	S	S	S	R	S	R	S	S	S	S	S	S	4	0	12
6.	1.00	R	8	R	S	R	R	R	S	I	S	S	S	S	S	S	S	S	5	1	10
7.	1.00	S	3	R	S	S	R	R	S	S	S	S	S	S	S	S	S	S	3	0	13
8.	1.00	R	32	R	R	R	R	R	S	S	S	S	S	S	S	S	S	S	6	0	10

FOX—cefoxitin; P—penicillin; CIP—ciprofloxacin; CN—gentamicin; E—erythromycin; DA—clindamycin; QD—quinupristine-dalfopristine; TE—tetracycline; TGC—tigecycline; C—chloramphenicol; FD—fusidic acid; LZD—linezolid; RA—rifampin; VA—vancomycin; DPC—daptomycin; SXT—cotrimoxazole; R—resistance; I—intermediate susceptibility; S—susceptibility.

**Table 3:** Characteristics of *S. aureus* strains isolated from skin lesions.

**No.**	**MIC of geranium essential oil** **[µL/mL]**	**Susceptibility to antibiotics**																	**Total**		
		FOX MIC [µg/mL]		P	CIP	CN	E	DA	QD	TE	TGC	C	FD	LZD	RA	VA	DPC	SXT	R	I	S
1.	0.25	S	3	R	S	S	S	S	S	S	S	S	S	S	S	S	S	S	1	0	15
2.	1.00	S	3	R	S	S	S	S	S	S	S	S	S	S	S	S	S	S	1	0	15
3.	1.00	S	2	R	S	S	S	S	S	S	S	S	S	S	S	S	S	S	1	0	15
4.	1.00	S	1.5	R	S	S	S	S	S	S	S	S	S	S	S	S	S	S	1	0	15
5.	0.25	S	3	R	S	S	S	S	S	S	S	S	S	S	S	S	S	S	1	0	15
6.	1.25	S	3	R	S	S	S	S	S	S	S	S	S	S	S	S	S	S	1	0	15
7.	1.50	R	32	R	S	S	R	R	S	S	S	S	S	S	S	S	S	S	4	0	12
8.	1.00	R	12	R	S	R	R	R	S	S	S	S	S	S	S	S	S	S	5	0	11
9.	1.00	R	12	R	S	S	R	R	S	S	S	R	S	S	S	S	S	S	5	0	11

FOX—cefoxitin; P—penicillin; CIP—ciprofloxacin; CN—gentamicin; E—erythromycin; DA—clindamycin; QD—quinupristine-dalfopristine; TE—tetracycline; TGC—tigecycline; C—chloramphenicol; FD—fusidic acid; LZD—linezolid; RA—rifampin; VA—vancomycin; DPC—daptomycin; SXT— cotrimoxazole; R—resistance; S—susceptibility.

**Table 4:** Characteristics of *S. aureus* strains isolated from postoperative wounds.

**No.**	**MIC of geranium essential oil** **[µL/mL]**	**Susceptibility to antibiotics**																	**Total**		
		FOX MIC [µg/mL]		P	CIP	CN	E	DA	QD	TE	TGC	C	FD	LZD	RA	VA	DPC	SXT	R	I	S
1.	1.00	S	3	R	S	S	S	S	S	S	S	S	S	S	S	S	S	S	1	0	15
2.	1.25	S	3	S	S	S	S	S	S	S	S	S	S	S	S	S	S	S	0	0	16
3.	2.25	R	8	R	S	S	S	S	S	R	S	S	S	S	S	S	S	S	3	0	13
4.	2.25	S	3	S	S	S	S	S	S	S	S	S	S	S	S	S	S	S	0	0	16
5.	1.00	S	3	R	S	S	R	R	S	S	S	S	S	S	S	S	S	S	3	0	13
6.	0.75	S	3	S	S	S	R	R	S	S	S	S	S	S	S	S	S	S	2	0	14
7.	1.50	R	8	R	S	S	R	R	S	R	S	R	S	S	S	S	S	S	6	0	10
8.	0.50	R	48	R	S	S	S	S	S	S	S	S	S	S	S	S	S	S	2	0	14

FOX—cefoxitin; P—penicillin; CIP—ciprofloxacin; CN—gentamicin; E—erythromycin; DA—clindamycin; QD—quinupristine-dalfopristine; TE—tetracycline; TGC—tigecycline; C—chloramphenicol; FD—fusidic acid; LZD—linezolid; RA—rifampin; VA—vancomycin; DPC—daptomycin; SXT— cotrimoxazole; R—resistance; S—susceptibility.

**Table 5:** Characteristics of *S. aureus* strains isolated from intubation tubes.

**No.**	**MIC of geranium essential oil** **[µL/mL]**	**Susceptibility to antibiotics**																	**Total**		
		FOX MIC [µg/mL]		P	CIP	CN	E	DA	QD	TE	TGC	C	FD	LZD	RA	VA	DPC	SXT	R	I	S
1.	0.25	S	3	R	S	S	S	S	S	S	S	S	S	S	S	S	S	S	1	0	15
2.	0.25	S	3	R	S	S	S	S	S	S	S	S	S	S	S	S	S	S	1	0	15
3.	1.00	R	96	R	R	S	R	R	S	S	S	S	S	S	S	S	S	S	5	0	11
4.	0.25	S	2	R	S	S	S	S	S	S	S	S	S	S	S	S	S	S	1	0	15
5.	1.50	R	24	R	S	S	S	S	S	S	S	S	S	S	S	S	S	S	2	0	14
6.	1.50	S	2	S	S	S	S	S	S	S	S	S	S	S	S	S	S	S	0	0	16
7.	0.25	R	16	R	S	S	R	R	S	S	S	S	S	S	S	S	S	S	4	0	12
8.	0.25	R	24	R	S	S	S	S	S	S	S	S	S	S	S	S	S	S	2	0	14
9.	0.75	R	24	R	S	S	R	R	S	S	S	S	S	S	S	S	S	S	4	0	12
10.	0.75	R	48	R	S	S	S	S	S	S	S	S	S	S	S	S	S	S	2	0	14
11.	2.50	R	8	R	S	S	R	R	S	R	S	R	S	S	S	S	S	S	6	0	10
12.	0.50	R	256	R	R	R	R	R	S	R	S	S	S	S	S	S	S	S	7	0	9
13.	1.50	R	128	R	R	S	R	R	S	S	S	S	S	S	S	S	S	S	5	0	11
14.	1.50	R	8	R	S	S	S	S	S	S	S	S	S	S	S	S	S	S	2	0	14

FOX—cefoxitin, P—penicillin; CIP—ciprofloxacin; CN—gentamicin; E—erythromycin; DA—clindamycin; QD—quinupristine-dalfopristine; TE—tetracycline; TGC—tigecycline; C—chloramphenicol; FD—fusidic acid; LZD—linezolid; RA—rifampin; VA—vancomycin; DPC—daptomycin; SXT— cotrimoxazole; R—resistance; S—susceptibility.

**Table 6:** Characteristics of *S. aureus* strains isolated from the conjunctival sack.

**No.**	**MIC of geranium essential oil** **[µL/mL]**	**Susceptibility to antibiotics**																	**Total**		
		FOX MIC [µg/mL]		P	CIP	CN	E	DA	QD	TE	TGC	C	FD	LZD	RA	VA	DPC	SXT	R	I	S
1.	0.25	R	32	R	S	S	S	S	S	S	S	S	S	S	S	S	S	S	2	0	14
2.	0.75	S	3	S	S	S	S	S	S	S	S	S	S	S	S	S	S	S	0	0	16
3.	0.75	S	3	R	S	S	S	S	S	S	S	S	S	S	S	S	S	S	1	0	15
4.	1.50	S	1.5	R	S	S	S	S	S	S	S	S	S	S	S	S	S	S	1	0	15
5.	1.00	S	2	S	S	S	R	R	S	S	S	S	S	S	S	S	S	S	2	0	14
6.	1.00	R	12	R	S	S	R	R	S	S	S	S	S	S	S	S	S	S	4	0	12

FOX—cefoxitin; P—penicillin; CIP—ciprofloxacin; CN—gentamicin; E—erythromycin; DA—clindamycin; QD—quinupristine-dalfopristine; TE—tetracycline; TGC—tigecycline; C—chloramphenicol; FD—fusidic acid; LZD—linezolid; RA—rifampin; VA—vancomycin; DPC—daptomycin; SXT— cotrimoxazole; R—resistance; S—susceptibility.

**Table 7:** Characteristics of *S. aureus* strains isolated from the throat.

**No.**	**MIC of geranium essential oil** **[µL/mL]**	**Susceptibility to antibiotics**																	**Total**		
		FOX MIC [µg/mL]		P	CIP	CN	E	DA	QD	TE	TGC	C	FD	LZD	RA	VA	DPC	SXT	R	I	S
1.	1.00	S	3	R	S	S	S	S	S	R	S	S	S	S	S	S	S	S	2	0	14
2.	0.25	S	1.5	S	S	S	S	S	S	S	S	S	S	S	S	S	S	S	0	0	16
3.	2.25	S	3	S	S	S	S	S	S	S	S	S	S	S	S	S	S	S	0	0	16
4.	1.00	S	3	S	S	S	S	S	S	S	S	S	S	S	S	S	S	S	0	0	16
5.	1.50	S	2	R	S	S	S	S	S	R	S	S	S	S	S	S	S	S	2	0	14
6.	0.75	S	3	S	S	R	S	S	S	S	S	S	S	S	S	S	S	S	1	0	15
7.	1.00	R	12	R	S	S	R	R	S	S	S	S	S	S	S	S	S	S	4	0	12
8.	0.75	S	3	R	S	S	S	S	S	S	S	S	S	S	S	S	S	S	1	0	15
9.	2.50	R	12	R	S	S	R	R	S	S	S	S	S	S	S	S	S	S	4	0	12
10.	2.50	R	32	R	S	R	S	S	S	R	S	S	S	S	S	S	S	S	4	0	12
11	1.00	R	48	R	S	S	S	S	S	R	S	S	S	S	S	S	S	S	3	0	13

FOX—cefoxitin; P—penicillin; CIP—ciprofloxacin; CN—gentamicin; E—erythromycin; DA—clindamycin; QD—quinupristine-dalfopristine; TE—tetracycline; TGC—tigecycline; C—chloramphenicol; FD—fusidic acid; LZD—linezolid; RA—rifampin; VA—vancomycin; DPC—daptomycin; SXT— cotrimoxazole; R—resistance; S—susceptibility.

**Table 8:** Characteristics of *S. aureus* strains isolated from stools.

**No.**	**MIC of geranium essential oil** **[µL/mL]**	**Susceptibility to antibiotics**																	**Total**		
		FOX MIC [µg/mL]		P	CIP	CN	E	DA	QD	TE	TGC	C	FD	LZD	RA	VA	DPC	SXT	R	I	S
1.	0.25	S	3	R	S	S	S	S	S	S	S	S	S	S	S	S	S	S	1	0	15
2.	0.25	S	3	R	S	S	R	R	S	S	S	S	S	S	S	S	S	S	3	0	13
3.	0.25	S	3	R	S	S	S	S	S	S	S	S	S	S	S	S	S	S	1	0	15
4.	0.25	R	64	R	R	S	R	R	S	S	S	S	S	S	S	S	S	S	5	0	11
5.	1.00	S	3	R	S	S	S	S	S	S	S	S	S	S	S	S	S	S	1	0	15
6.	0.75	S	2	R	S	S	S	S	S	S	S	S	S	S	S	S	S	S	1	0	15
7.	2.25	S	3	S	S	S	S	S	S	S	S	S	S	S	S	S	S	S	0	0	16
8.	2.00	S	3	S	S	S	S	S	S	S	S	S	S	S	S	S	S	S	0	0	16
9.	0.75	S	3	S	S	S	S	S	S	S	S	S	S	S	S	S	S	S	0	0	16
10.	1.00	S	3	R	S	S	S	S	S	S	S	S	S	S	S	S	S	S	1	0	15
11.	1.25	S	3	R	S	S	S	S	S	S	S	S	S	S	S	S	S	S	1	0	15
12.	1.50	S	2	R	S	S	R	R	S	S	S	S	S	S	S	S	S	S	3	0	13
13.	2.50	R	16	R	S	S	S	S	S	S	S	S	S	S	S	S	S	S	2	0	14
14.	1.00	R	32	R	S	S	S	S	S	S	S	S	S	S	S	S	S	S	2	0	14

FOX—cefoxitin; P—penicillin; CIP—ciprofloxacin; CN—gentamicin; E—erythromycin; DA—clindamycin; QD—quinupristine-dalfopristine; TE—tetracycline; TGC—tigecycline; C—chloramphenicol; FD—fusidic acid; LZD—linezolid; RA—rifampin; VA—vancomycin; DPC—daptomycin; SXT—cotrimoxazole; R—resistance; S—susceptibility.

### 2.3. Susceptibility of the Clinical *S. aureus* Strains to the Geranium Oil

**Figure 1:**
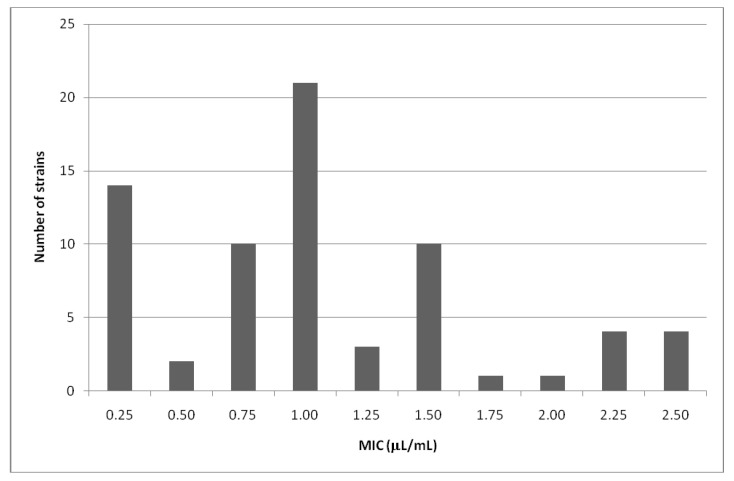
Susceptibility of the clinical *S. aureus* strains to the geranium oil.

The geranium oil obtained from *Pelargonium graveolens* Ait. shows a very strong activity against the standard *S. aureus* strain (ATCC 433000) and also against the examined *S. aureus* strains obtained from the clinical materials. The values of MIC against clinical *S. aureus* strains ranged from 0.25 µL/mL to 2.5 µL/mL. The growth of the standard *S. aureus* strain, ATCC 433,000, was inhibited by 0.25 µL/mL of the tested oil. The majority of *S. aureus* strains studied: 47 out of 70, were sensitive to the oil concentrations of 1.00 µL/mL or lower (Figure 1). These strains were isolated from: nasal cavity 6/8, skin lesions 7/9, postoperative wounds 4/8, intubation tubes 9/14, conjunctival sack 5/6, throat 7/11 and stools 9/14 (Table 2 and Table 8). 

In our study the geranium oil had a strong antimicrobial activity against clinical *S. aureus* strains with different mechanisms of resistance. The lowest values of geranium essential oil MICs (0.25 µL/mL and 0.5 µL/mL) inhibited the growth of both MRSA strains exhibiting high MICs for cefoxitin (16–256 µg/mL) and MSSA isolates presenting the lowest MICs for cefoxitin (1.5–3 µg/mL) (Table 5). However, MRSA strains exhibiting high MICs for cefoxitin (12–32 µg/mL) were also inhibited in 1.75 µL/mL and 2.50 µL/mL of the tested oil (Table 2, Table 7 and Table 8). No correlation between MICs for geranium oil and MICs for cefoxitin could be noticed. 

The most effective against MRSA and MSSA clinical *S. aureus* strains were concentrations: 1.00 µL/mL (n = 21), 0.25 µL/mL (n = 14), 0.75 µL/mL (n = 10) and 1.50 µL/mL (n = 10) (Table 9).

**Table 9:** Resistance to cefoxitin in correlation with MICs of geranium oil among *S. aureus* strains.

Number of strains	MIC of geranium oil [µL/mL]									
	0.25	0.50	0.75	1.00	1.25	1.50	1.75	2.00	2.25	2.50
MRSA	4	2	3	10	0	6	1	0	1	4
MSSA	10	0	7	11	3	4	0	1	3	0
Total	14	2	10	21	3	10	1	1	4	4

The effect of the geranium oil on *S. aureus* strains exhibiting MLS_B_ mechanism of drug resistance was also analysed. The growth of most such bacterial isolates was inhibited also in the presence of 1.00 µL/mL (n = 11), 0.25 µL/mL (n = 4) and 1.50 µL/mL (n = 4) of the tested oil (Table 10).

**Table 10:** MLS_B_ resistance in correlation with MICs of geranium oil among *S. aureus* strains.

Number of strains with different MLS_B_ mechanisms	MIC of geranium oil [µL/mL]									
	0.25	0.50	0.75	1.00	1.25	1.50	1.75	2.00	2.25	2.50
Inducible MLS_B_	3	1	3	5	0	1	1	0	0	0
Constitutive MLS_B_	1	0	0	6	0	2	0	0	0	2
Constitutive and inducible MLS_B_	0	0	0	0	0	1	0	0	0	0
Total	4	1	3	11	0	4	1	0	0	2

Finally, the effect of the geranium oil on *S. aureus* strains resistant to antibiotics other than β-lactams, macrolides and lincosamides was analysed. The growth of most bacterial strains resistant to tetracycline, gentamicin, and ciprofloxacin was inhibited by the lower concentrations of the geranium oil (≤1.50 µL/mL).

## 3. Discussion

Because of increasing concern over *S. aureus* resistance to beta-lactam and glycopeptide antibiotics, especially among MRSA strains, new therapeutic options are being sought. The ‘golden age’ of antibiotics has gone. Attempts are made to modify cephalosporins, carbapenems, glycopeptides, quinolones, tetracyclines and other antibiotics to obtain compounds with new activity against staphylococci. Only a few examples of antimicrobial agents administered against difficult to treat staphylococcal infections may be mentioned, *i.e.*, linezolid, quinupristine-dalfopristine, tigecycline, glycopeptides (dalbavancin, telavancin, oritavancin), telithromycin, daptomycin, and ceftobiprole [18,19,20]. High costs of the therapies using the drugs listed above as well as the many side-effects associated with their pharmacokinetics should incline us to look for other alternatives. In our opinion geranium oil may be one of them. We have determined the susceptibility to this essential oil among 70 *S. aureus* strains isolated from the nasal cavity, conjunctival sack, throat, skin lesions, postoperative wounds, intubation tubes, and from stools. The tested strains exhibited various antibiotic susceptibility patterns (Table 2–Table 8). Resistance to methicillin was found in 44.3% of all bacterial isolates. The percentage of MLS_B_-positive *S. aureus* strains amounted to 37.1%. Susceptibility of both multidrug resistant and fully susceptible clinical isolates to the tested oil was investigated. All *S. aureus* strains proved to be susceptible to tigecycline, rifampin, trimethoprim-sulphamethoxazole, linezolid, fusidic acid, quinupristine-dalfopristine, vancomycin, and daptomycin. 

The obtained results, in accordance with the literature, show that geranium oil has antimicrobial properties against all tested strains. The activity is due to the high content of alcoholic compounds with antibacterial properties such as citronellol and geraniol, which account for over 40% of the ingredients of the geranium oil [21,22].

The geranium oil obtained from *Pelargonium graveolens* Ait. shows a very strong activity against the standard *S. aureus* strain (ATCC 433000) and also against the examined *S. aureus* strains coming from clinical materials. Moreover, the effectiveness of the geranium oil in relation to multidrug resistant *S. aureus* strains, even those resistant to β-lactam antibiotics and those showing macrolide-lincosamide-streptogramin resistance (MLS_B_ resistance), was revealed in our study. The values of MIC against clinical *S. aureus* strains ranged from 0.25 µL/mL to 2.5 µL/mL. The growth of the standard *S. aureus* strain, ATCC 433000, was inhibited by 0.25 µl/mL of the tested oil. 

According to the literature, geranium oil showed growth inhibitory effect against methicillin-resistant* S. aureus* ssp. *aureus* ATCC 700699 using the disk-diffusion method (30 μL of tested oil per 13 mm disk) with an inhibition zone of 26 mm [23]. Prabuseenivasan *et al*. [24] reported that oil obtained from *Pelargonium graveolens* Ait. used at concentrations higher than 12.8 mg/mL inhibited the growth of the *S. aureus* standard strain ATCC 25923. In our investigations geranium oil was active against *S. aureus* ATCC 433000 at a much lower concentration of 0.25 µL/mL. Research on antimicrobial properties of geranium oil alone and in combination with tea tree oil demonstrated its very strong activity against non-typable MRSA strains and MRSA strains belonging to phage type 15 isolated from the wounds of burn patients in a burns unit. The results of these experiments show that geranium oil can be used in the treatment of MRSA infections which have created major problems for burn units and also intensive care units [25]. Malik and Sink [26] in their investigations demonstrated very good efficiency of the geranium oil against antibiotic sensitive and resistant bacterial strains isolated from urinary tract infections of significant bacteriuria. *S. aureus* sensitive and resistant to kanamycin, ampicillin and ciprofloxacin were used to check antibacterial activity of the geranium oil by the disk-diffusion method using 10 μL of the oil per 6 mm disk. The inhibition zone for *S. aureus* (sensitive) was 26.5 mm and for *S. aureus* (resistant)—26.3 mm. The antibiotic sensitive and resistant strains of *S. aureus* were inhibited at 8.96 mg/mL of the geranium oil. In our research, multidrug resistant clinical strains isolated from different clinical materials were mainly sensitive to 1.00 µL/mL or lower concentrations of geranium oil and even the most resistant strains were inhibited at 2.50 μL/mL.

In our investigation it has been found that geranium essential oil is effective against *S. aureus* strains with different mechanisms of drug resistance. Geranium oil can be applied not only in the treatment of dysentery, urinary tract and skin infections, but also in inflammation of the mouth, larynx, pharynx caused by bacterial and fungal pathogens. It can be used as an effective air disinfectant and as an additive to antiseptic preparations, and be used in the hospitals, nursing homes and clinics. The application of essential oils in the treatment of human diseases, particularly infectious diseases caused by multidrug resistant bacterial strains, may be an interesting alternative to synthetic drugs. Synergy of action essential oils with antibiotics and chemotherapeutics presents an opportunity for significant reductions of therapeutic doses, reduction of the adverse effects of antibiotic therapy and prevention of antibiotic-resistant strain formation. Because of the therapeutic problems associated with particularly resistant strains, essential oils can be useful in fighting against the microflora provoking hospital-acquired infections. Doran *et al.* [27] have tested the antibacterial activity of geranium and lemongrass essential oils against MRSA strains alone and in combination. The tests shown that essential oil vapours inhibited the growth of antibiotic-sensitive and antibiotic-resistant bacteria, but the effects were variable, depending on the exposure time and testing environment. Bearden *et al.* [28] showed the antibacterial effectiveness of a combination of bezetonin chloride and volatile oils from tea tree or thyme against MRSA strains isolated from wounds. Karpanen *et al.* [29] showed that essential oils from tea tree eucalyptus and thyme, together with chlorhexidine digluconate, an antiseptic used in dermatology, were effective against *S. epidermidis* pathogenic strains and proved thyme to be the most effective. It shows not only inhibitory properties against the investigated bacteria but also prevents biofilm formation. Concerning the application of oils, many studies have shown that essential oils are well absorbed by nasal, oral, gastric, intestinal mucous membranes and the skin. The active compounds in the oils are incorporated into cell membranes and influence enzyme and ion channel function as well as receptor proteins. Oils are not accumulated in the human body and are neutralized by binding to glucuronic acid and eliminated with urine [30]. The LD_50 _values for most essential oils are greater than 5 g/kg body weight, while therapeutic doses are usually only a few drops per day. Details—monographs of oils have been published in National Pharmacopeia, National Pharmacopeia, ISO, WHO and Council of Europe, to ensure the availability of the necessary information about essential oils: their source, concentration of active components, and therapeutic doses. The parameters of geranium oil are clarified in ISO-4731. Although plant medicines are considered to be safe, the possibility of overdoses and their interactions with synthetic drugs administered orally must be considered. Investigations on essential oils’ mechanism of action and their components are currently being carried out both *in vitro* and *in vivo* [31,32], but many products containing essential oils have been already patented. Due to their antimicrobial properties they are used in the treatment of respiratory, digestive system, and also in skin and oral infections. A number of essential oils have been identified as effective antibacterials used in food to control natural spoilage processes and to prevent the growth of microorganisms and also as additives to drugs and cosmetics [33].

## 4. Experimental

### 4.1. Essential Oil Analysis

A commercial essential oil from *Pelargonium graveolens* Ait. was purchased from the manufacturer (POLLENA-AROMA Poland) and analyzed by GC-FID-MS in the Institute of General Food Chemistry, Lodz University of Technology, using a Trace GC Ultra apparatus (Thermo Electron Corporation) with FID and MS DSQ II detectors and FID-MS splitter (SGE). Operating conditions: apolar capillary column Rtx-1ms (Restek), 60 m × 0.25 mm i.d., film thickness 0.25 µm; temperature program, 50–300 °C at 4 °C/min; SSL injector temperature 280 °C; FID temperature 300 °C; split ratio 1:20; carrier gas helium at a regular pressure 200 kPa; FID temperature 260 °C; carrier gas, helium; 0.5 mL/min; split ratio 1:20. Mass spectra were acquired over the mass range 30–400 Da, ionization voltage 70 eV; ion source temperature 200 °C.

Identification of components was based on the comparison of their MS spectra with those in a laboratory-made MS library, commercial libraries (NIST 98.1, Wiley Registry of Mass Spectral Data, 8th Ed. and MassFinder 4) and with literature data [34,35] along with the retention indices on an apolar column (Rtx-1, MassFinder 4) associated with a series of alkanes with linear interpolation (C_8_-C_26_). A quantitative analysis (expressed as percentages of each component) was carried out by peak area normalization measurements without correction factors.

### 4.2. Antibacterial Activity of Essential Oil

For testing of antibacterial activity a *S. aureus* ATCC 433000 strain that came from collection of Medical and Sanitary Microbiology Department, Medical University of Lodz, was used. The clinical *S. aureus* strains, collected in 2010, came from different materials taken from patients of five hospital wards (internal medicine, surgical ward, otolaryngology, gastroenterology, and intensive care unit) of the Polish Mother's Health Center in Lodz, Poland. Bacterial strains were isolated from the nasal cavity, conjunctival sack, throat, skin lesions, postoperative wounds, intubation tubes, and stools. *S. aureus* isolates involved in this study came from the patients requiring antistaphylococcal antibiotic therapy due to the active infections (this applies to the strains isolated from the conjunctival sack, throat, skin lesions, postoperative wounds, and intubation tubes) or the patients that were *S. aureus* carriers (applies to the isolates from the nasal cavity and feces). The standard and clinical strains were cultivated on Columbia agar medium and incubated at 37 °C for 48 h in aerobic conditions. Bacterial suspensions with an optical density of 0.5 on a McFarland scale were prepared. A BioMérieux densitometer was used.

The essential oil was diluted in ethanol. This solution was mixed with an agar medium to obtain concentrations from 0.125 to 2.5 µL/mL and poured into Petri dishes [36]. Inoculum containing 1.5 × 10^8^ CFU/mL (0.1 mL) per spot was seeded upon the surface of agar with various oil concentrations, as well as upon that with no oil added (strain growth control). The MICs values were determined after 24 h of incubation at 37 °C under aerobic conditions. The analysis of the antibacterial activity of the oil was independently performed three times. Control media containing ethanol (at concentrations used in the dilutions) did not inhibit the growth of bacterial strains.

### 4.3. Antibiotic Susceptibility of the Clinical *S. aureus* Strains

Antimicrobial susceptibility testing was performed in accordance with the criteria of the European Committee on Antimicrobial Susceptibility Testing (EUCAST) [37]. The disk-diffusion method was used to investigate the bacterial susceptibility to 14 antimicrobial agents: cefoxitin (FOX, 30 μg, Oxoid), erythromycin (E, 15 μg, Oxoid), clindamycin (DA, 2 μg, Oxoid), tetracycline (TE, 30 μg, Oxoid), tigecycline (TGC, 15 μg, Bio-Rad), chloramphenicol (C, 30 μg, Bio-Rad), ciprofloxacin (CIP, 5 μg, Oxoid), rifampin (RA, 5 μg, Bio-Rad), gentamicin (CN, 10 μg, Oxoid), trimethoprim-sulphamethoxazole (SXT, 1.25/23.75 μg, Oxoid), linezolid (LZD, 30 μg, Oxoid), fusidic acid (FD, 10 μg, Oxoid), and quinupristine-dalfopristine (QD, 15 μg, Oxoid); additionally susceptibility to penicillin (P, 10 IU, Oxoid) was also tested. The following cut-off values for the disk-diffusion determination of resistance (R) and susceptibility (S) have been used: cefoxitin (R < 22, S ≥ 22), penicillin (R < 26, S ≥ 26), erythromycin (R < 18, S ≥ 21), clindamycin (R < 19, S ≥ 22), tetracycline (R < 19, S ≥ 22), tigecycline (R < 18, S ≥ 18), chloramphenicol (R < 18, S ≥ 18), ciprofloxacin (R < 20, S ≥ 20), rifampin (R < 23, S ≥ 26), gentamicin (R < 18, S ≥ 18), trimethoprim-sulphamethoxazole (R < 14, S ≥ 17), linezolid (R < 19, S ≥ 19), fusidic acid (R < 24, S ≥ 24), quinupristine-dalfopristine (R < 18, S ≥ 21), according to EUCAST criteria [33]. Susceptibility to vancomycin (VA256, BioMérieux) and daptomycin (DPC256, BioMérieux) was carried out using the E-test method. This technique was also used to determine MIC value for cefoxitin (FX256, BioMérieux). The following cut-off values for the E-test determination of resistance have been used: cefoxitin (R > 4 µg/mL), vancomycin (R > 2 µg/mL), daptomycin (R ≥ 1 µg/mL) [37]. Inducible and constitutive resistance to macrolide-lincosamide-streptogramin B (MLS_B_ resistance) was investigated using a double disk technique with erythromycin (15 μg) and clindamycin (2 μg). Bacterial isolates were inoculated on Mueller-Hinton II Agar (bioMerieux) and incubated in an aerobic atmosphere at 35 °C for 18 h (24 h for cefoxitin and vancomycin).

## 5. Conclusions

1. Geranium oil obtained from *Pelargonium graveolens* Ait. was active at concentrations of 0.25–2.50 μL/mL against multidrug resistant staphylococci of different origin.
2. No correlation was found between MICs values for geranium oil inhibiting the growth of *S. aureus* strains and MICs values for cefoxitin.
3. The growth of most MLS_B_-positive *S. aureus* strains was inhibited in lower MICs values of the tested oil.

## References

[1] DeLeo F.R., Otto M., Kreiswirth B.N., Chambers H.N. (2010). Community-associated methicillin-resistant *Staphylococcus aureus*. Lancet.

[2] Rafee Y., Abdel-Haq N., Asmar B., Salimnia T., Pharm C.V., Rybak P.M.J., Amjad M. (2012). Increased prevalence of methicillin-resistant *Staphylococcus aureus* nasal colonization in household contacts of children with community acquired disease. BMC Infect. Dis..

[3] Wang J.T., Liao C.H., Fang C.T., Chie W.C., Lai M.S., Lauderdale T.L., Chang S.C. (2010). Incidence of and risk factors for community-associated methicillin-resistant *Staphylococcus aureus* acquired infection or colonization in intensive-care-unit patients. J. Clin. Microbiol..

[4] Bigos M., Denys A. (2008). The MRSA hospital infections. Int. Rev. Allergol. Clin. Immunol..

[5] Boers S.A., van Ess I., Euser S.M., Jansen R., Tempelman F.R., Diederen B.M. (2011). An outbreak of a multiresistant methicillin-susceptible *Staphylococcus aureus* (MR-MSSA) strain in a burn centre: The importance of routine molecular typing. Burns.

[6] Loomba P.S., Taneja J., Mishra B. (2010). Methicillin and vancomycin resistant *S. aureus* in hospitalized patients. J. Glob. Infect. Dis..

[7] Warren R. (2012). *Staphylococcus aureus*—A cross sectional study of prevalence and risk factors in one general practice. Aust. Fam. Physician.

[8] Cheng V.C., Chan J.F., Lau E.H., Yam W.C., Ho S.K., Yau M.C., Tse E.Y., Wong A.C., Tai J.W., Fan S.T. (2011). Studying the transmission dynamics of meticillin-resistant *Staphylococcus aureus* in Hong Kong using spa typing. J. Hosp. Infect..

[9] Kim S.P., Shah N.D., Karnes R.J., Weight C.J., Frank I., Moriarty J.P., Han L.C., Borah B., Tollefson M.K., Boorjian S.A. (2012). The implications of hospitalacquired adverse events on mortality, length of stay and costs for patients undergoing radical cystectomy for bladder cancer. J. Urol..

[10] Edris A.E. (2007). Pharmaceutical and therapeutic potentials of essential oils and their individual volatile constituents: A review. Phytother. Res..

[11] Bakkali F., Averbeck S., Averbeck D., Idaomar M. (2008). Biological effect of essential oils—A review. Food Chem. Toxicol..

[12] Silva N.C.C., Fernandes Júnior A. (2010). Biological properties of medicinal plants: A review of their antimicrobial activity. J. Venom. Anim. Toxins Incl. Trop. Dis..

[13] Reichling J., Schnitzler P., Suschke U., Saller R. (2009). Essential oils of aromatic plants with antibacterial, antifungal, antiviral and cytotoxic properties—An oview. Forsch. Komplementment..

[14] Lis-Balchin M., Peter K.V. (2004). Geranium. Hand Book of Herbs and Species.

[15] Verma R.K., Laiq-ur-Rahman, Verma R.S., Kalra A., Kukreja A.K., Bisht A.S., Chauhan A., Khanuja S.P.S. (2011). Assessing N-use efficiency, planting time and economics of fertilizer N in rose-scented geranium (*Pelargonium graveolens* L’ Herit) in Western Himalayan Region of India. Afr. J. Agric. Res..

[16] Shawl A.S., Kumar T., Chishi N., Shabir S. (2006). Cultivation of rose scented *Geranium* (*Pelargonium* sp.) as a cash crop in Kasmir Valley. Asian J. Plant Sci..

[17] Rosato A., Vitali C., De Laurentiis N., Armenise D., Antonietta Milillo M. (2007). Antibacterial effect of some essential oils administered alone or in combination with Norfloxacin. Phytomedicine.

[18] Ramirez P., Fernández-Barat L., Torres A. (2012). New therapy options for MRSA with respiratory infection/pneumonia. Curr. Opin. Infect. Dis..

[19] Fernandez J., Abbanat D., Shang W., He W., Amsler K., Hastings J., Queenan A.M., Melton J.L., Barron A.M., Flamm R.K. (2012). Synergistic activity of ceftobiprole and vancomycin in a rat model of infective endocarditis caused by methicillin-resistant and glycopeptide-intermediate *Staphylococcus aureus*. Antimicrob. Agents Chemother..

[20] Vilhena C., Bettencourt A. (2012). Daptomycin: A review of properties, clinical use, drug delivery and resistance. Mini-Rev. Med. Chem..

[21] Lis-Balchin M., Deans S.G., Hart S. (2007). Bioactive *Geranium* oils from different commercial sources. J. Essent. Oil Res..

[22] Fabio A., Cermelli C., Fabio G., Nicoletti P., Quaglio P. (2007). Screening of the antibacterial effects of a variety of essential oils on microorganisms responsible for respiratory infections. Phytother. Res..

[23] Chao S., Young G., Oberg C., Nakaoka K. (2008). Inhibition of methicillin-resistant *Staphylococcus aureus* (MRSA) by essential oils. Flavour Fragr. J..

[24] Prabuseenivasan S., Jayakumar M., Ignacimuthu S. (2006). *In vitro* antibacterial activity of some plant essential oils. BMC Complement. Altern. Med..

[25] Edwards-Jones V., Buck R., Shawcross S.G., Dawson M.M., Dunn K. (2004). The effect of essential oils on methicillin-resistant *Staphylococcus aureus* using a dressing model. Burns.

[26] Malik T., Singh P. (2010). Antibacterial effects of essentials oils against uropathogens with varying sensitivity to antibiotics. Asian J. Biol. Sci..

[27] Dorian A.L., Morden W.E., Dunn K., Edwards-Jones V. (2009). Vapour-phase activities of essential oils against antibiotic sensitive and resistant bacteria including MRSA. Lett. Appl. Mcrobiol..

[28] Bearden D.T., Allen G.P., Christensen J.M. (2008). Comparative *in vitro* activities of topical wound care products against community-associated methicillin-resistant *Staphylococcus aureus*. J. Antimicrob. Chemother..

[29] Karpanen T.J., Worthington T., Hendry E.R., Conway B.R., Lambert P.A. (2008). Antimicrobial efficacy of chlorhexidine digluconate alone and in combination with eucalyptus oil, tea tree oil and thymol against planktonic and biofilm cultures of *Staphylococcus epidermidis*. J. Antimicrob. Chemother..

[30] Vercesi A.E., Kowaltowski A.J., Grijalba M.T., Meinicke A.R., Castilho R.F. (1997). The role of the reactive oxygen species in mitochondrial permeability transition. Biosci. Rep..

[31] Schmitt S., Schaefer U.F., Doebler L., Reichling J. (2009). Cooperative interaction of monoterpenes and phenylpropanoids on the *in vitro* human skin permeation of complex composed essential oils. Planta Med..

[32] Ohkawara S., Tanaka-Kagawa T., Furukawa Y., Nishimura T., Jinno H. (2010). Activation of the human transient receptor potential vanilloid subtype 1 by essential oils. Biol. Pharm. Bull..

[33] Sienkiewicz M., Kowalczyk E., Wasiela M. (2012). Recent patents regarding essential oils and the significance of their constituents in human health and treatment. Recent Pat. Anti-Infect. Drug Discov..

[34] Adams R.P. (2007). Identification of Essential Oil Components by Gas Chromatography/Mass Spectroscopy.

[35] Joulain D., König W.A. (1998). The Atlas of Spectral Data of Sesquiterpene Hydrocarbons.

[36] Kalemba D., Kunicka A. (2003). Antibacterial and antifungal properties of essential oils. Curr. Med. Chem..

[37] European Committee on Antimicrobial Susceptibility Testing (EUCAST) Breakpoint Tables for Interpretation of MICs and Zone Diameters, version 2.0.

